# The role of FGF19–FGFR4 signaling pathway in liver health and disease: Guardian or destroyer

**DOI:** 10.1016/j.gendis.2025.101989

**Published:** 2025-12-19

**Authors:** Zhangfan Wu, Yijun Wang, Jiaqian Zhang, Siwen Li, Junjie Wen, Dian Hu, Junqing Jiang, Zerui Zhang, Xiangyuan Luo, Limin Xia

**Affiliations:** Department of Gastroenterology, Institute of Liver and Gastrointestinal Diseases, Hubei Key Laboratory of Hepato-Pancreato-Biliary Diseases, Tongji Hospital of Tongji Medical College, Huazhong University of Science and Technology, Wuhan, Hubei 430030, China

**Keywords:** Fibroblast growth factor 19, Fibroblast growth factor receptor 4, Hepatocellular carcinoma, Liver disease, Metabolic dysfunction-associated steatotic liver disease

## Abstract

The fibroblast growth factor (FGF) 15/19–FGF receptor (FGFR) 4 signaling pathway is a crucial endocrine regulatory pathway within the FGF family. The FGF15/19–FGFR4 signaling pathway plays multiple key roles in the liver, involving core physiological processes such as metabolic regulation, bile acid homeostasis maintenance, and hepatocyte proliferation and repair, and is also closely related to the pathogenesis of various liver diseases. At present, targeted therapeutic strategies for the FGF19–FGFR4 signaling axis have shown significant therapeutic potential. Agonists that simulate the physiological functions of FGF19 have been proven to effectively regulate bile acid and lipid metabolism in metabolic diseases and improve liver steatosis and fibrosis. Meanwhile, drugs that selectively inhibit FGFR4 have also demonstrated positive anti-tumor activity in specific tumor types driven by FGF19 overexpression. Given the crucial role of FGF19–FGFR4, clarifying the key mechanisms of this pathway in both physiology and pathology, as well as summarizing targeted therapy, is of vital importance. This review highlights the key role of FGF15/19–FGFR4 signaling in regulating liver physiological functions and reveals how its abnormal expression contributes to the occurrence of benign and malignant liver diseases. In addition, this review points out the potential of FGF15/19–FGFR4 signaling as a biomarker in different liver diseases and briefly discusses the existing treatment strategies for this signaling pathway.

## Introduction

Fibroblast growth factors (FGFs) and their transmembrane receptors are involved in the regulation of liver development, metabolism, and regeneration. The mammalian FGF family comprises 23 members. Except for FGF11–14, which are non-secreted but share sequence homology with other FGFs, the remaining FGFs can be secreted into the extracellular space and function in an autocrine, paracrine, or endocrine manner.^1^ The fibroblast growth factor receptor (FGFR) family consists of five members, FGFR1–5. Among them, FGFR1–4 possess intrinsic tyrosine kinase activity and can activate downstream signaling pathways such as mitogen-activated protein kinase (MAPK) and phosphatidylinositol 3-kinase (PI3K)–protein kinase B (AKT) by specifically binding to FGFs, thereby regulating cell proliferation, differentiation, and metabolic homeostasis. In contrast, FGFR5 lacks kinase activity.^2^

As the central regulator of systemic metabolism, the liver maintains energy homeostasis through coordinated regulation of lipid catabolism, glycogen storage, and bile acid (BA) synthesis. Critically, the FGF19–FGFR4 signaling axis orchestrates hepatic metabolic responses by precisely modulating BA homeostasis, thereby exerting a profound influence on systemic energy balance. The particularity and importance of FGF19–FGFR4 signaling in the liver are determined by their unique expression and binding pattern. The terminal motif of FGF19 exhibits a robust binding affinity with FGFR4, enhancing receptor activation and downstream signaling.^3^ Moreover, FGFR4, unlike FGFR1–3, does not undergo alternative splicing in its ligand-binding domain (D3), resulting in a high affinity for its cognate ligand, FGF19 ^4^. β-Klotho (KLB) is an essential coreceptor for FGF15/19 binding to FGFR4. In contrast to the widely distributed FGFRs, KLB has a limited expression pattern *in vivo*, mainly in metabolically important tissues, including the liver, pancreas, and white adipose tissue.^3^ Consequently, the liver stands out as the sole organ where both FGFR4 and KLB exhibit increased expression levels concurrently. FGFR4 mediates almost all FGF19 activities in the liver.^3^

Extensive evidence links FGF19–FGFR4 signaling to liver function, and its disruption is associated with chronic liver conditions and hepatocellular carcinoma (HCC). This review systematically summarizes the mechanisms underlying FGF19–FGFR4 signaling in liver physiological function, inflammatory progression, and tumorigenesis. In addition, we emphasize the principal difficulties associated with the clinical application of FGF19–FGFR4 signaling modulation and suggest potential approaches to surmount these challenges.

## General overview of FGF19 and FGFR4: Structure and expression

FGF19 is a constituent of the FGF19 subfamily, initially discovered in the human fetal brain in 1999.^5^ It is located on chromosome 11q13.3, and FGF15 is its mouse FGF ortholog.^4^^,^^5^ Characteristic of the FGF family, FGF19 includes a conserved central domain that comprises around 120 to 130 amino acids. This domain is structured by 12 antiparallel β-sheets.^6^ The transduction of FGF–FGFR signaling typically relies on the assistance of heparan sulfate. This cofactor enhances signal transmission by forming stable complexes with FGFs and FGFRs on the target cell surface. However, FGF19 and its subfamily members possess a unique topological structure at their heparan sulfate-binding sites, resulting in low affinity for heparan sulfate.^7^ This reduced affinity underlies the endocrine nature of FGF19, distinguishing it from paracrine FGFs. Instead of relying on heparan sulfate, FGF19 requires KLB, a single-pass transmembrane co-receptor lacking intrinsic kinase activity, to bind and activate FGFR4.^8^^,^^9^ KLB stabilizes the FGF19–FGFR4 complex, improves signaling efficiency, and promotes systemic metabolic regulation.^7^ FGF19 signaling is not limited to FGFR4. In the presence of KLB, it can also activate other receptors, such as FGFR1c, particularly in peripheral tissues like adipose tissue.^10^^,^^11^ Activation of the FGF19–FGFR1c pathway significantly contributes to systemic metabolic improvements, including enhanced insulin sensitivity and increased energy expenditure.^12^^,^^13^

FGFR4 is the principal receptor for FGF19 and the sole FGFR expressed in hepatocytes of adults.^8^ It is located on chromosome 5q35.1 and spans approximately 11 kb. Comprising 802 amino acids,^14^ FGFR4 has a relative molecular mass ranging from 95 to 110 kDa, depending on its glycosylation status.^15^ FGFR4 consists of three domains: an extracellular immunoglobulin-like domain (D1, D2, D3), a domain that spans the cell membrane, and an internal region that functions as a tyrosine kinase.^6^ Located between D1 and D2 is an acid box, a sequence rich in serine that helps modulate the inhibitory role of FGFR4.^16^ The D2 and D3 domains are pivotal in attaching to FGFs and facilitate the dimerization of the receptor, a critical event for initiating subsequent signaling mechanisms.^17^

## Physiological function of FGF15/19–FGFR4

**Figure 1:**
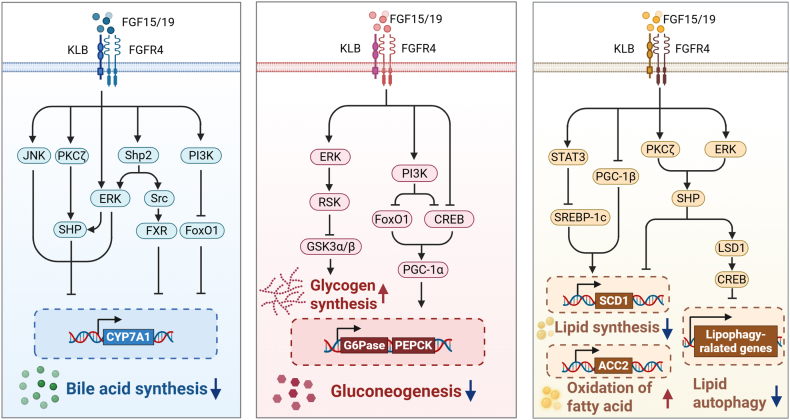
The function of FGF15/19–FGFR4 in hepatocytes. FGF15/19 in the terminal ileum is transported to the hepatocyte surface via the portal circulation and exerts its physiological effects by binding to FGFR4. The green represents bile acid (BA) metabolism, the pink represents glucose metabolism, and the orange represents lipid metabolism.

FGF15/19–FGFR4 primarily regulates biometabolic activities such as BA synthesis, lipid metabolism, glycogen synthesis, and gluconeogenesis (Fig. 1).

## BAs regulation

BAs are a class of amphiphilic molecules derived from cholesterol. They are mainly synthesized in the liver and are the main functional components of bile. BAs are produced in the liver, stored in the gallbladder, and released into the small intestine after meals. They play a key role in fat digestion, fat-soluble vitamin absorption, cholesterol metabolism regulation, and signal transduction.^7^ Due to their detergent-like properties, their synthesis must be tightly regulated to avoid tissue toxicity.^18^

FGF15/19 secreted by the intestine negatively regulates hepatic BA metabolism in a unique manner. FGF15 circulates through the portal vein or lymphatics, binding with FGFR4 on the surface of hepatic cells, triggering the activation of FGFR substrate 2α (FRS2α), and consequently activating the extracellular regulated protein kinase (ERK) signaling pathways.^19^ ERK increases the stability of small heterodimer partner (SHP).^20^ SHP is also activated by protein kinase C zeta (PKCζ).^21^ This cascade inhibits the enzymatic activity of cholesterol 7α-hydroxylase (CYP7A1), leading to a reduction in BA synthesis and the mediation of negative feedback regulation of BAs. Nevertheless, FRS2α can also bypass the FGF19–FGFR4–MAPK/ERK pathway and directly activate ERK through phosphorylation of the non-receptor tyrosine phosphatase Shp2 and the non-receptor tyrosine kinase Src.^22^ Simultaneously, activated Src can phosphorylate the Tyr residue Y67 of FXR, which has an important effect on the nuclear localization and DNA-binding of FXR.^22^ In addition, the FGF15/19–FGFR4 signaling pathway suppresses CYP7A1 through decreasing hepatic forkhead transcription factor 1 (FoxO1) activity in a PI3K-dependent phosphorylation manner.^23^ The down-regulation of CYP7A1 expression has also been demonstrated through FGF19–FGFR4–Jun N-terminal kinase (JNK) signaling.^24^ These early studies provided crucial insights into the pivotal role of FGF19–FGFR4 in the intricate regulation of BA metabolism.

The gallbladder is also critical for the homeostatic regulation of BA metabolism. FGF15 knockout mice showed intermittent gallbladder dysfunction, while recombinant FGF15 or FGF19 treatment increased wild-type gallbladder volume and normalized function in knockout mice.^25^ Similarly, FGFR4 knockout mice and KLB gene knockout mice both exhibited a smaller gallbladder volume.^26^^,^^27^ These findings collectively underscore the broader impact of the FGF19–FGFR4–KLB pathway on gallbladder dynamics and BA homeostasis. Paradoxically, FGF15/19 retains the ability to induce gallbladder relaxation in FGFR4 knockout mice.^28^ Thus, while the pathway’s importance in gallbladder volume maintenance is well-supported, its precise role in BA release regulation requires further mechanistic clarification.

## Glucose and lipid metabolism

FGF15/19–FGFR4 signaling is a key regulator of glucose and lipid homeostasis. Early seminal work by Tomlinson et al first demonstrated its metabolic impact: transgenic mice expressing FGF15 exhibited reduced adiposity, improved plasma metabolite profiles, and resistance to diet-induced obesity effects attributed to increased energy expenditure.^29^ Crucially, unlike insulin signaling, which promotes adipogenesis, FGF15/19–FGFR4 signaling uniquely drives glycogen anabolism while suppressing lipogenesis, thereby reducing risks of metabolic disorders.^30^ This distinct mechanism positions FGF19 as a promising therapeutic target for metabolic diseases.

In animals fasted overnight, FGF19 induces the phosphorylation of glycogen synthase kinases, glycogen synthase kinase 3 alpha (GSK3α, Ser21) and glycogen synthase kinase 3 beta (GSK3β, Ser9), which are negative regulators of glycogen synthesis. This leads to increased enzyme glycogen synthase activity and liver glycogen content.^30^ Mice deficient in FGF15 exhibit an over 50% reduction in hepatic glycogen levels and impaired glucose uptake from the bloodstream, fully restored by FGF19 administration.^30^ Mechanistically, FGF15/19 represses gluconeogenesis-associated gene expression by promoting the dephosphorylation of the cAMP regulatory element binding protein (CREB).^31^ The reduced CREB activity results in decreased expression of peroxisome proliferator-activated receptor γ coactivator-1α (PGC-1α) and other genes involved in hepatic metabolism.^31^ FGF15/19-FGFR4 inhibited FoxO1 via PI3K, as mentioned above, resulting in the down-regulation of key enzymes in gluconeogenesis, such as phosphoenolpyruvate carboxykinase (PEPCK) and glucose-6-phosphatase (G6Pase).^23^ FGF15/19 was also reported to be released from the intestine to promote hepatic lipid metabolism, adapting to increased dietary lipids. Under high-fat feeding conditions, mice lacking endogenous FGF15 show significant lipid metabolism impairment compared with wild-type mice, manifested by hepatic steatosis and exacerbated ER stress.^32^ Conversely, FGF19 supplementation can protect liver cells from lipid accumulation-induced apoptosis, promote regeneration of the steatosis liver, reduce body weight and obesity, and lower circulating glucose and lipid levels.^32^^,^^33^ Mechanism studies have shown that FGF15/19 can suppress late-state postprandial liver fat production through several epigenetic signals.^34^^,^^35^ FGF19 inhibits lipogenesis by suppressing sterol regulatory element-binding protein 1c (SREBP-1c) activity through alterations in signal transducer and activator of transcription 3 (STAT3) and peroxisome proliferator-activated receptor-γ coactivator 1β (PGC-1β) activity.^36^ FGF19–FGFR4–SHP signaling promotes DNA methylation modification of lipid-expressing genes.^35^ FGF19-FGFR4 signaling down-regulates the adipogenic enzyme stearoyl CoA desaturase 1 (SCD1) and increases fatty acid oxidation by decreasing acetyl CoA carboxylase 2 (ACC2) activity.^6^ Additionally, activated SHP recruits histone demethylase LSD1 after 4 h of feeding, mediates the demethylation of H3K4me2/3, to epigenetically repress CREB-bound lipophagy-related genes.^34^

## FGF15/19–FGFR4 as a metabolic integrator

The FGF15/19–FGFR4 signaling pathway serves as a core integrator of hepatic metabolic programs during the transition between fed and fasted states. It maintains metabolic homeostasis through three primary physiological functions: regulating BA balance by inhibiting excessive synthesis to prevent intrahepatic accumulation and injury; modulating postprandial lipid metabolism by promoting fatty acid oxidation and suppressing *de novo* lipogenesis; and stabilizing blood glucose by enhancing glycogen synthesis and inhibiting gluconeogenesis, thereby reducing postprandial glucose fluctuations.

The activity of the FGF15/19–FGFR4 pathway exhibits distinct circadian characteristics. Serum FGF19 levels peak 90–120 min after postprandial BA elevation, a rhythm that disappears during fasting,^37^ indicating that FGF19 serves as a key temporal signal linking periodic feeding to feedback inhibition of BA synthesis. Further studies reveal complexities in this synchronization: conjugated and unconjugated BAs display divergent enterohepatic cycling rhythms. Postprandial conjugated BAs drive rhythmic FGF19 secretion, while the nocturnal peak of unconjugated BAs reflects intrinsic circadian activity of the gut microbiota, independent of FGF19 regulation.^38^ Additionally, the gallbladder, as an important source of FGF19, contributes to systemic rhythmicity—its removal alters the FGF19 peak and increases BA synthesis,^38^ underscoring its role in circadian regulation. At the molecular level, FGF15/19 interacts extensively with core circadian components. Clinical observations indicate that night-shift work disrupts both plasma FGF19 levels and nicotinamide adenine dinucleotide-dependent sirtuin 1 (SIRT1) signaling, while animal studies show that *Fgf15* knockout disrupts the expression of circadian-related genes, including SIRT1, in the liver.^39^ These findings suggest that the FGF15/19–FGFR4 pathway may form a feedback loop with core clock proteins such as SIRT1 to maintain metabolic circadian integrity.

The FGF15/19–FGFR4 pathway also coordinates crosstalk among multiple metabolic processes. Its activation induces SHP expression, which simultaneously suppresses the rate-limiting BA enzyme CYP7A1 and lipogenic enzymes,^36^^,^^40^ thereby reducing the accumulation of endogenous and exogenous lipids and excessive BA production, and alleviating metabolite toxicity. Furthermore, the activation of FoxO1 by the FGF15/19–FGFR4 pathway inhibits both BA synthesis and PGC-1α-driven gluconeogenesis,^23^ contributing to glycemic stability while limiting exogenous carbon input. Owing to its multi-target regulatory capacity over BA, glucose, and lipid metabolism, the FGF19–FGFR4 pathway demonstrates considerable potential for the treatment of metabolic diseases, offering a strategy for synergistic intervention and holistic metabolic improvement.

In summary, the FGF19–FGFR4 pathway functions not only as a key negative regulator of BA synthesis but also as a crucial temporal integrator that unifies nutritional signals, gut microbiota activity, gallbladder function, and hepatic metabolism within a circadian framework, thereby precisely coordinating the body’s energy metabolic balance.

## The upstream regulation of FGF15/19

**Figure 2:**
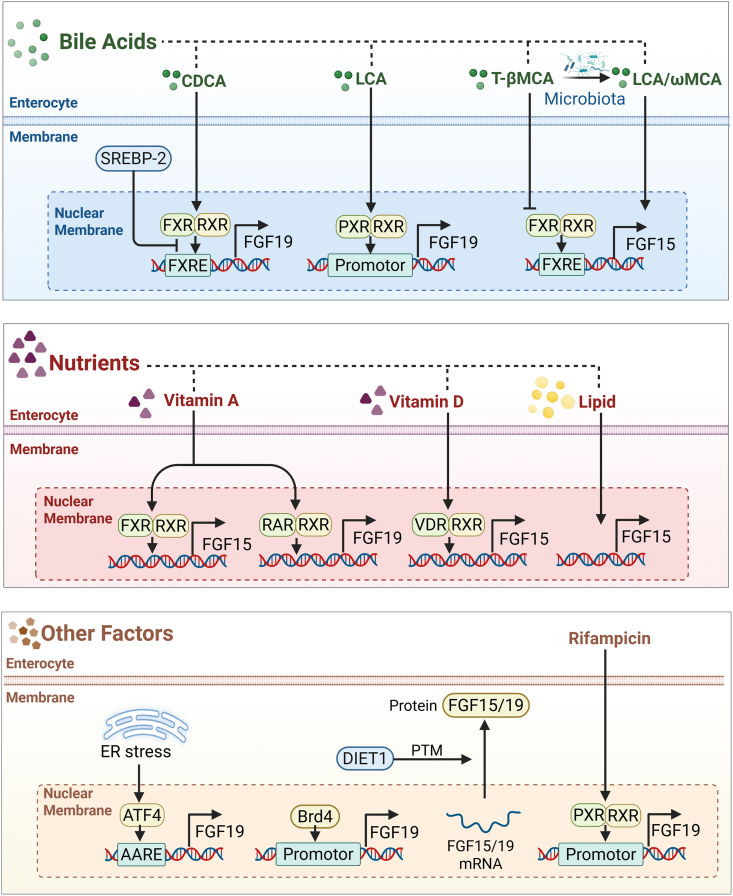
The secretion of FGF15/19. Bile acids (BAs), nutrients, and other intra- and extracellular factors influence the secretion of FGF15/19 by enterocytes (see text for further details).

Intestinal FGF15/19 expression is regulated by various factors (Fig. 2). In 2003, Jason et al discovered the first functional farnesoid X receptor responsive element (FXRE) in the FGF19 gene’s second intron, the binding site for farnesoid X receptor (FXR).^24^ Subsequently, Takeshi Inagaki et al observed that FXR activation provoked the secretion of FGF15 in mouse intestinal epithelium.^40^ A reporter assay conducted using the human colon cancer cell line LS174T revealed the presence of three FXREs located within the 5′-flanking region of the FGF19 gene, extending up to 8.8 kb. FXR binds to these FXREs as an FXR/retinoid X receptor (RXR) heterodimer, which promotes the expression of FGF19.^41^ Activated sterol regulatory element-binding protein 2 (SREBP-2) binds directly to FXR and inhibits FXR binding to FXRE.^42^

The secretion level and composition of BAs affect the FGF15/19 expression. In a human-derived enterocyte cell line, chenodeoxycholic acid (CDCA) was able to strongly induce FGF19 expression by binding to the FXR/RXR heterodimer.^24^ In LS174T cells, secondary BA lithocholic acid (LCA) significantly induced FGF19 promoter activity in combination with the pregnane X receptor (PXR)/RXR heterodimer.^43^ In mice, a mouse-specific primary BA, tauro-conjugated β-muricholic acid (T-βMCA), which is an antagonist of FXR, was able to be converted to the secondary BAs LCA and ω-muricholic acid (ωMCA) in the presence of gut microbes, thereby elevating FGF15 expression.^44^

In addition, macronutrients and micronutrients modulate FGF15/19 expression. Lipids and acute or chronic cholesterol feeding increased ileal FGF15 expression in mice.^45^ The induction of FGF15 by vitamin D is mediated through the RXR/vitamin D receptor (VDR) heterodimer, whereas the induction of FGF15 by vitamin A is mediated through the RXR/FXR heterodimer.^46^ However, in the human intestine, vitamin A binds to the retinoic acid receptor (RAR)/RXR heterodimer to induce FGF19 expression.^47^

Some components within the enterocyte also affect FGF15/19 levels. In 2013, the DIET1 protein was first identified as being able to affect FGF15/19 levels at both the mRNA and post-transcriptional stages.^48^ Knockdown of the DIET1 gene resulted in decreased FGF15 levels and reduced FGF19 protein secretion.^49^ A DIET1 coding variant (rs12256835) causes an H1721Q amino acid substitution that increases FGF19 protein secretion.^50^ Overall, genetic variation in DIET1 may be one of the determinants of the extent of FGF15/19 expression. During endoplasmic reticulum (ER) stress, FGF19 mRNA expression can be induced. This phenomenon was detected in the human colorectal adenocarcinoma cell line Caco-2, which is dependent on the binding of activating transcription factor 4 (ATF4). ATF4 is a transcription factor activated under ER stress and binds to the functional amino acid response element (AARE) within the FGF19 promoter region.^51^ Bromodomain-containing protein 4 (Brd4) in enterocytes, as a transcriptional regulator that binds to acetylated histones, also binds to the FGF19 promoter region to up-regulate FGF19 expression.^52^ Finally, some drugs like the potent PXR ligand rifampicin significantly induce FGF19 promoter activity in enterocytes.^43^

In addition to the intestine, large amounts of FGF19 mRNA have been observed in the human gallbladder and common bile duct.^53^ However, the specific modalities that regulate FGF19 expression outside of enterocytes require further study. Besides, the mechanisms underlying the regulation of FXR–FGF15/19 signaling, such as intestinal perfluorobutyric acid intake and bariatric surgery, remain largely unknown.^54^^,^^55^

## FGF15/19–FGFR4 dysregulation in non-neoplastic hepatopathy

**Figure 3:**
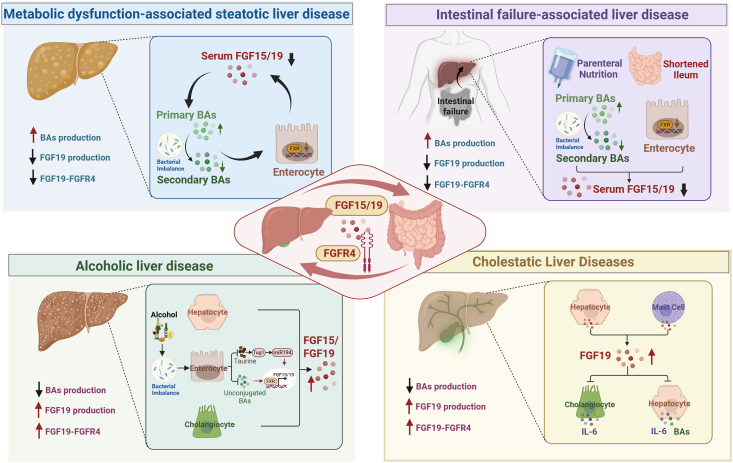
The role of FGF19–FGFR4 varies in different liver diseases, impacting disease progression. The reduction in the proportion of FXR agonistic bile acids (BAs) and the decrease in FXR expression in enterocytes are the primary causes for the diminished FGF19 production in metabolic dysfunction-associated steatotic liver disease (MASLD). Subsequently, the attenuated FGF19–FGFR4 signaling in the liver plays a series of injurious roles in the progression of liver disease. In intestinal failure-associated liver disease, in addition to the aforementioned factors, parenteral nutrition and a shortened ileum also contribute to reduced FGF19 expression. In alcoholic liver disease (ALD), although alcohol consumption reduces FGF19 expression in a FXR-dependent manner, hepatocytes and cholangiocytes compensatorily express FGF19, exerting a protective effect. In cholestatic liver diseases, FGF19 secretion by mast cells (MCs) has also been observed, which inhibits the secretion of IL-6 by cholangiocytes and hepatocytes, ameliorating liver inflammation.

Given the central role of the FGF15/19–FGFR4 axis in preserving hepatic metabolic balance, its dysregulation is increasingly recognized as a common pathogenic mechanism underlying a range of liver disorders, including metabolic dysfunction-associated steatotic liver disease (MASLD), alcoholic liver disease (ALD), cholestatic liver diseases, and intestinal failure-associated liver disease (Fig. 3). Elucidating the precise molecular mechanisms of this pathway and exploring targeted therapeutic interventions are therefore of considerable importance for both basic research and clinical translation.

## MASLD

As one of the most common chronic liver diseases, NAFLD is a series of liver diseases, including simple nonalcoholic fatty liver without evidence of hepatocyte injury, nonalcoholic steatohepatitis (NASH) with hepatocyte injury and inflammation, fibrosis, and eventually cirrhosis, and even HCC.^56^ In 2023, NAFLD was renamed as MASLD. MASLD refers to a clinical syndrome characterized by hepatic steatosis combined with one or more cardiovascular metabolic risk factors, while excluding other causes of hepatic steatosis.^57^ Dysregulation of the FGF15/19–FGFR4 signaling pathway in MASLD can lead to disease progression.

## Pathogenesis

A clinical investigation has revealed that in normal individuals, non-NASH NAFLD patients, and NASH patients, the plasma concentration of FGF19 decreases in a gradient.^58^ This makes the activation of FGF19–FGFR4 signaling significantly impaired in the liver. Changes in the total level and proportion of BA in NAFLD patients reduce the secretion of FGF19. There are notable distinctions in the distribution of BAs between NASH, nonalcoholic fatty liver, and the normal liver. Puri et al have noticed that NASH patients demonstrated a significant rise in primary BAs and a significant decline in secondary BAs.^59^ Mechanistically, a high-fat diet leads to changes in intestinal flora, which affects the process of BA metabolism.^60^ The primary BAs and secondary BAs in the BA pool have opposing effects on the nuclear receptor FXR in the intestinal cells. Zhu et al showed that in NAFLD patients, the percentage of secondary BA deoxycholic acid (DCA), which uniquely appears to be increased as an antagonistic ligand for FXR, may play a dominant role in increasing the expression of FGF19.^61^ FGF19–FGFR4 is an important negative feedback signal for BA synthesis. When the FGF19–FGFR4 signal is weakened, increased levels of principal BAs have been significantly linked to advanced stages of fat accumulation in liver cells, inflammation within lobules, inflammation in the portal areas, and the swelling of liver cells.^59^ In addition to reducing the activation of FGFR4 by directly reducing FGF19 levels, other ways in which BAs regulate the binding of FGF19–FGFR4 in hepatocytes are still unclear. KLB determines the efficiency of FGF19 binding to FGFR4, and it has been shown that in pediatric patients with biopsy-proven NAFLD, those with reduced KLB expression variants have an increased risk of hepatic lobular inflammation, hepatocellular ballooning, and liver fibrosis.^62^ The activation of FGF19–FGFR4–SHP is impaired in NAFLD patients, resulting in reduced methylation of DNMT3A-dependent SREBP-1-regulated lipogenic genes^35^ In conclusion, in the liver of patients with MASLD, the FGF19–FGFR4 signaling pathway is impaired, lipogenic genes are overexpressed, BA synthesis is increased, and fatty acid β-oxidation is reduced.

## Targeted therapeutic strategies

The FGF19–FGFR4 signaling may be advantageous in managing MASLD. This signal can be targeted at multiple levels to fulfill targeted therapies. One method to activate FGF19 expression is by activating FXR, an upstream signal in enterocytes. Gut flora can alter the BA pool, which is an important regulator of FXR. In rodent models of MASLD, probiotic supplementation markedly elevates FXR and FGF15 expression, enhances gut microbial diversity, reduces the prevalence of harmful bacterial species, and ultimately mitigates the progression of MASLD.^63^ As for FXR agonists, there are currently obeticholic acid (OCA), silibinin, and tropifexor (LJN452) coming into the picture. Oral silibinin enhances FXR transcription by decreasing HDAC2 activity and selectively promotes FGF15/19 expression in the ileum.^64^ OCA increases cholic acid (CA) and CDCA while decreasing taurodeoxycholic acid and tauroursodeoxycholic acid in murine models of NASH. This results in hepatic reactive oxygen species (ROS) accumulation, lipid peroxidation, and altered iron metabolism, activating hepatic stellate cells.^65^^,^^66^ Clinical trials have shown that OCA can improve fibrosis and alleviate symptoms in patients with NASH.^67^^,^^68^ The potential drawback of OCA is its tendency to cause dose-dependent adverse effects, such as pruritus, constipation, diarrhea, and hyperlipidemia, and to increase the risk of gallstone formation.^67^^,^^69^^,^^70^ The degree of pruritus can be positively correlated with the patient’s serum interleukin-31 (IL-31) level,^71^ which can be attributed to the off-target activation of G protein-coupled BA receptor 1 (GPBAR1) by OCA.^72^ In the NASH mouse model, tropifexor treatment increased FGF15 levels and proved more effective than OCA, despite being used at a lower dose (less than 1 mg/kg versus 25 mg/kg). Tropifexor significantly reversed hepatic fibrosis, reduced steatohepatitis, and lowered NAFLD scores, hepatic triglycerides, and profibrogenic gene expression.^72^ Currently, researchers are also developing other FXR agonists such as nidufexor (LMB763)^73^ and compound 42.^74^

Furthermore, there is a potential avenue for exploration: the creation of FGF19 analogs. Aldafermin (NGM282 or M70), the most thoroughly researched analog of FGF19 to date, has undergone clinical trials for various liver diseases, extending beyond MASLD. Activation of FGF19–FGFR4 signaling in hepatocytes results in decreased adipogenesis and increased lipolysis. Aldafermin was observed to rapidly and significantly reduce liver fat content in NASH patients.^75^^,^^76^ It showed the ability to improve liver fibrosis in NASH patients with or without cirrhosis,^77^^,^^78^ and the activity of liver disease was also significantly reduced during 12 weeks of treatment.^79^ The predominant genus composition of NASH patients treated with aldafermin remained essentially unchanged. There was a significant and dose-dependent increase in the number of the rare genus Veillonella, which is capable of degrading lactate.^80^ Alafermin mainly inhibits glycine-coupled BAs, but not taurine-coupled BAs. Glycine-coupled BAs have high hydrophobicity and cytotoxicity.^81^ Diarrhea was the most common adverse reaction, and the incidence of diarrhea increased with higher drug doses.^77^^,^^78^ Despite these therapeutic advantages, aldafermin’s oncogenic potential remains a critical concern. Preclinical models reveal that even this engineered analog cooperates with MYC overexpression to drive aggressive hepatocarcinogenesis in mutation-prone livers.^82^ Critically, FGFR4 overactivation poses oncogenic risks even without genetic drivers like MYC: in murine NASH models with FGF21 knockout, unrestricted signaling induced hepatocyte proliferation and epithelial-to-mesenchymal transition..^83^ This phenotype aligns with FGF19–FGFR4 pathway hyperactivation observed in human HCC tissues,^84^ confirming conserved pro-tumorigenic mechanisms. To reconcile the metabolic efficacy and oncogenic risks of FGF19 analogs, three independent strategies are proposed: First, developing pathway-selective analogs engineered to suppress CYP7A1 while avoiding activation of RAS–MAPK/β-catenin pathways. Second, deploying receptor-independent combinations such as ASBT inhibitors with FGF15, which reduces hepatic fat by 70% without FGFR4 engagement.^85^ Third, precision modulation of FGFR4 downstream signaling. Tissue-targeted delivery systems enable hepatocyte-specific FGFR4 activation, thereby restricting exposure to mutation-prone biliary epithelia and reducing off-target oncogenic risks.

## ALD

ALD is a common condition in Europe and the U.S., with a daily intake exceeding 40 g of alcohol significantly increasing the risk of alcoholic fatty liver among the majority of people.^86^ Down-regulation of FGF15–FGFR4 signaling was observed in the ALD mouse model. Ethanol treatment in non-cirrhotic rats led to a notable decrease in FGF15 levels.^87^

## Pathogenesis

In patients with ALD and alcoholic steatohepatitis (ASH), circulating FGF19 levels are markedly elevated compared with healthy individuals.^88^^,^^89^ Notably, in ALD, FGF19 is not only derived from enterocytes but also ectopically expressed by hepatocytes, especially cholangiocytes and ductal progenitor cells.^89^ This dysregulation reflects a compensatory attempt to suppress hepatic BA synthesis via FGFR4-mediated CYP7A1 inhibition; however, paradoxically, total BA levels remain elevated due to impaired bile flow and hepatic BA deposition.^89^

Mechanistically, chronic ethanol consumption induces intestinal dysbiosis, leading to increased levels of unconjugated BAs. This is attributed to elevated cholylglycine hydrolase and BA deconjugating enzymes in the gut microbiota.^90^ The excess unconjugated BAs can suppress intestinal FXR activity, thereby down-regulating FGF15/19 expression. In parallel, ethanol-induced dysbiosis reduces intestinal taurine levels, which further inhibits FXR signaling through the taurine-up-regulated gene 1 (Tug1)–microRNA194 (miR194) axis, resulting in increased hepatic BA synthesis via reduced FGF15/19–FGFR4 signaling.^91^

Experimental studies further support this duality: in mouse models of acute and chronic alcohol intake, exogenous FGF19 overexpression markedly reduced secondary BA levels and disrupted gut microbial balance, unexpectedly promoting overgrowth of pathogenic bacteria and exacerbating hepatic inflammation.^92^ The exact mechanism underlying this paradoxical inflammatory response remains unclear and warrants further investigation. Despite these insights, our understanding of the FGF15/19–FGFR4 axis in ALD pathogenesis remains limited. Elucidating its molecular dynamics in the context of ethanol-induced gut-liver crosstalk will be essential for the development of targeted therapies.

## Targeted therapeutic strategies

The level of serum FGF19 can serve as a biomarker to assess the severity of ALD. A higher concentration indicates a more serious condition.^89^ FXR agonists that promote FGF19 expression in ALD-related clinical trials have been carried out, including OCA and INT-787. The clinical trial of OCA in patients with moderately severe alcoholic hepatitis was terminated prematurely due to reports of its hepatotoxicity (NCT02039219). The clinical trial of INT-787 in severe alcohol-associated hepatitis is ongoing (NCT05639543). In mouse experiments, oral administration of Lactobacillus rhamnosus GG (LGG)-derived exosome-like nanoparticles improved intestinal flora and inhibited miR194 expression, thereby restoring suppressed FXR-FGF15 signaling.^91^ There is an ongoing clinical trial investigating the function of LGG supplementation in alcoholic hepatitis and alcohol use disorder (NCT05178069). In mice, a high-soluble dietary fiber diet increased the abundance of *Bacteroides acidifaciens* and attenuated ALD. Bile salt hydrolase produced by *Bacteroides acidifaciens* activates FXR–FGF15 signaling, while FGF15 enhances the expression of ornithine aminotransferase in hepatocytes. This process helps convert accumulated ornithine into glutamate, promoting the detoxification of ammonia.^85^ These results provide a new perspective on the treatment of ALD.

## Cholestatic liver diseases

Cholestatic liver disorders, resulting from cholestasis-induced liver damage and fibrosis, are typified by alterations in the gut microbiota and an overabundance of hepatotoxic BAs.^93^ Primary biliary cholangitis (PBC) and primary sclerosing cholangitis (PSC) represent common forms of cholestatic liver diseases.^94^ FGF15/19-FGFR4 plays a crucial role in maintaining BA homeostasis, and its dysregulation contributes to the pathogenesis of cholestatic liver diseases.

## Pathogenesis

In mice with cholestatic liver disease modeled by bile duct ligation (BDL), hepatic concentrations of the FXR antagonist T-βMCA were markedly elevated, and hepatic concentrations of the FXR agonist CDCA appeared to be reduced.^93^ Liver injury induced by BDL in mice was associated with a reduction of *Lactobacillus acidophilus*. This resulted in reduced intestinal FXR–FGF15 signaling and increased hepatic BA production. *Lactobacillus acidophilus* supplementation improved hepatic cholestasis.^95^

However, in patients with cholestatic liver disease, the level of FGF19, which enterocyte FXR regulates, did not show a corresponding attenuation but was significantly elevated. Compared with normal subjects, elevated serum FGF19 and a concomitant decrease in *de novo* BA synthesis were observed in both PBC and PBC-autoimmune hepatitis overlap syndrome patients.^96^^,^^97^ This abnormally elevated FGF19 may be secreted by hepatocytes. FGF19 mRNA expression was almost undetectable in the normal liver, whereas in cholestatic patients, FGF19 mRNA was 31-fold–374-fold higher than in controls.^98^ Furthermore, alternative sources of FGF19 have been hypothesized, including mast cells (MCs) that infiltrate the hepatic and intestinal tissues. Mice with cholestatic liver disease develop hepatic MC infiltration, and experiments on BDL mice in 2021 showed that knockdown of MC down-regulated MC-FXR/FGF15 expression to attenuate ductular reaction, activation of hepatic stellate cells, and liver fibrosis, as well as intestinal inflammation.^99^ Numerous individuals with PSC are comorbid with inflammatory bowel disease, which usually precedes the onset of PSC.^100^ Compared with healthy individuals, PSC patients and patients with PSC and inflammatory bowel disease have increased serum FGF19 levels and SHP expression.^99^ The above findings further demonstrate the possibility of FGF19 production by MCs aggregated in inflammation.

In particular, reduced FXR staining was observed in cholangiocytes and hepatocytes in PBC patients who did not respond adequately to ursodeoxycholic acid.^101^ Rachel et al conducted a study where they co-cultured human cholangiocytes with CD4^+^ T cells to simulate the biliary stress response. The study found that FGF19 treatment significantly reduced cholangiocyte IL-6 release. This suggests that the increased IL-6 expression induced by a decreased FXR/FGF19 axis in cholangiocytes with hyperinflammation may be a potential mechanism for biliary tract injury in PBC.^101^ Cazzagon et al found that biliary acid toxicity decreases in PBS patients with an enlarged gallbladder, while severity increases in those who underwent cholecystectomy. Additionally, in a PSC mouse model, cholecystectomy led to increased cholangitis and hepatic fibrosis.^102^ Combined with the fact that FGF15-FGFR4 signaling enhances gallbladder storage capacity,^25^, ^26^, ^27^ as mentioned in the previous elaboration of the physiological functions of FGF15/19, it is, therefore, possible that down-regulation of FGF19–FGFR4 may exacerbate the severity of cholestatic disease through disturbances in the regulation of gallbladder diastole and contraction.

## Targeted therapeutic strategies

To mitigate the buildup of BAs, strategies must focus on both reducing their synthesis and enhancing their elimination from the body. Due to the issue of BA overproduction, several drugs currently in development for the treatment of MASLD are also undergoing clinical trials for cholestatic liver disease, such as aldafermin (NCT02704364) and cilofexor (NCT02808312). A phase II, double-blind, placebo-controlled trial assessed the efficacy of tropifexor (LJN452), an FXR agonist, in alleviating cholestatic signs in a randomized patient cohort with PBC, thereby endorsing its continued clinical investigation.^103^

In the multidrug resistance protein 2 knockout (Mdr2^−/−^) mouse model of PSC, intestinal FXR–FGF15/19 signaling was promoted by Glucagon-like peptide-2 (GLP-2) and led to a decrease in the expression of CYP7A1 and an increase in the expression of CYP2C70 in the liver.^104^ In addition, GLP-2 therapy not only ameliorated liver fibrosis in a PSC murine model but also exhibited anti-inflammatory properties in an inflammatory bowel disease murine model. This indicates that the co-administration of FXR agonists alongside GLP-2 analogs holds promise for the management of patients with PSC and inflammatory bowel disease.^104^

In BDL and Mdr2^−/−^ murine models, the administration of the probiotic LGG notably elevated serum and ileal levels of FGF15 and reduced the expression of hepatic CYP7A1 and BA synthesis. Concurrently, the gut microbiota composition was modified, leading to a rise in the ratio of unconjugated BAs and enhanced fecal and urinary BA excretion.^93^ Moreover, *ex vivo* investigations revealed that LGG reduced the suppressive impact of T-βMCA on the levels of FXR and FGF19 expression in Caco-2 cells.^93^ In mice, melatonin ameliorated cholestatic liver disease by reshaping the gut microbiota and activating the intestinal FXR-FGF15 axis-mediated inhibition of hepatic BA synthesis and promotion of BA excretion.^105^

Interestingly, BA synthesis is almost completely inhibited in advanced PSC, at which point agonizing the FXR/FGF19 axis is unlikely to be beneficial.^106^ FGF19 plays a more biomarker role at this time, and the higher the serum FGF19 level in patients, the worse the prognosis.

## Intestinal failure-associated liver disease

Long-term intestinal failure and parenteral nutrition have been found to cause a series of hepatobiliary complications, one of which is known as intestinal failure-associated liver disease. A multivariate analysis of chronic intestinal failure revealed that diminished FGF19 levels were correlated with persistent cholestasis. Furthermore, individuals exhibiting diminished FGF19 expression had a decreased 5-year survival rate (54% versus 66%).^107^

## Pathogenesis

Among pediatric patients with intestinal failure, a notable reduction in the prevalence of bacteria involved in BA transformation was observed in those exhibiting cholestasis, in contrast to those without, influencing the ratio of primary BAs to the total BA pool.^108^ At the same time, the expression of FXR in their ileum was reduced, serum FGF19 levels were significantly decreased, and the expression of CYP7A1 was significantly increased.^108^ In a retrospective study that enrolled 52 children with intestinal failure, Mutanen et al observed a positive correlation between FGF19 and the length of the remaining ileum.^109^ Serum levels of FGF19 were markedly reduced in pediatric patients with intestinal failure relative to healthy peers. Additionally, children who underwent complete ileal resection displayed lower FGF19 concentrations than those with partial preservation of the ileum. The authors also reported that serum FGF19 concentrations were lower in children currently receiving PN compared with those who had transitioned off PN. Although this retrospective study does not provide evidence, it indicates a correlation between FGF19 and intestinal failure-associated liver disease.^109^ A potential hypothesis is that partial loss of the ileum may impair the activity of the FXR–FGF19 axis, causing progressive liver injury. It warrants further laboratory research to substantiate this hypothesis.

## Targeted therapeutic strategies

Automated chyme reinfusion was able to up-regulate the intestinal FXR/FGF19 axis, resulting in decreased bile salt synthesis, normalization of gut microbes, and reduction of markers of liver injury and local intestinal inflammation.^110^ Xiao et al conducted experiments on mice and found that oral administration of antibiotics (gentamicin or vancomycin) reduced colonic microbial diversity. Additionally, primary BAs increased while secondary BAs, particularly the T-βMCA, decreased. This reduction affected the role of the FXR/FGF19 axis in BA metabolism, resulting in an inhibitory effect on BA accumulation.^108^ Through an upward adjustment of hepatic FXR signaling, the FXR agonist GW4064 protected mice against cholestasis associated with parenteral feeding.^111^ The potential role of FXR agonists in the treatment of intestinal failure-associated liver disease is illustrated by the up-regulation of canalicular bile, sterol, and phospholipid transporters, as well as the reduced activation and recruitment of macrophages.^111^

## FGF19–FGFR4 in HCC

Altered FGF19 and FGFR4 are prevalent in HCC, with FGF19 amplification in 15% of HCC, indicating poorer outcomes in liver cancer.^112^ FGF19 serves as a prognostic indicator for the early relapse of HCC and signifies an unfavorable outcome following curative hepatectomy.^113^ Ectopic expression of FGF19 in transgenic mice promotes the development of HCC by 10–12 months of age. In mice, FGF15 has been implicated in fibrosis-associated HCC development.^114^ Collectively, these results imply that interventions directed at the FGF19–FGFR4 pathway may offer a potential treatment approach for HCC. Moreover, the development of biomarkers to stratify patients with HCC based on FGF19–FGFR4 status could personalize treatment approaches and improve clinical outcomes. The translation of these insights into clinical practice will require rigorous preclinical and clinical evaluation to ensure the safety and efficacy of FGF19–FGFR4-targeted therapies in the management of HCC.

## Aberrant expression

FGF19 is located on chromosome 11q13.3 next to the crucial oncogene driver CCND1 in HCC. The amplification of 11q13.3 has been recognized as a common genetic alteration in HCC, present in approximately 5%–14% of cases, correlated with high expression levels and a poor prognosis.^115^ FGF19 and CCND1 are situated 45 kb apart, and their amplification consistently occurs simultaneously in HCC, leading to the up-regulation of both genes.^116^ FGF19 is posited as an etiologic factor in hepatocarcinogenesis.^116^^,^^117^ Generally, this amplification tends to appear at advanced stages in aggressive or proliferated HCC.^115^^,^^118^ The classification of HCC by molecular phenotype indicates that FGF19 amplification is strongly correlated with a specific subpopulation known as macrotrabecular-massive HCC, which is defined as having more than half of its size composed of macrotrabecular architecture.^119^^,^^120^ This subpopulation has an aggressive phenotype with distinct features, including high serum levels of alpha-fetoprotein, the G3 transcriptomic subgroup, TP53 mutations, and an immunosuppressive tumor microenvironment. Notably, it also shows increased neo-angiogenesis and vascular remodeling, as evidenced by elevated angiopoietin 2 and vascular endothelial growth factor A levels.^119^^,^^120^ Recently, several studies proved that macrotrabecular-massive HCC showed a great association with the poor outcomes of HCC patients using imaging techniques. The preoperative CT/MRI features could be recognized as a significant prognostic factor for early recurrence in patients with HCC or unfavorable outcomes after hepatic resection.^121^, ^122^, ^123^ A substantial amount of research is focusing on the significant role of FGF19-FGFR4 in hepatocarcinogenesis. In the following chapter, we will summarize our own and other researchers’ findings concerning the mechanism through which FGF19–FGFR4 promotes hepatocarcinogenesis.

## Effects on biological characters

**Figure 4:**
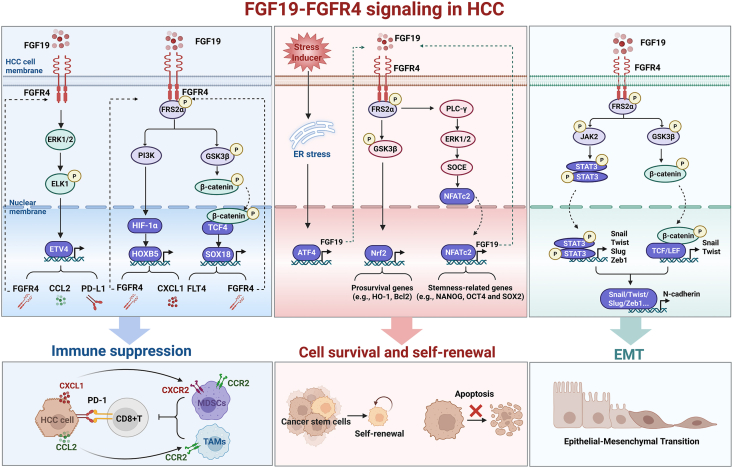
Effects of FGF19–FGFR4 on biological characteristics of hepatocellular carcinoma. Immune suppression, tumor growth, and regeneration, as well as epithelial-to-mesenchymal transition (EMT) progression, are all regulated by FGF19–FGFR4 signaling (see text for further details).

FGF19–FGFR4 signaling promotes liver cancer progression through multiple mechanisms. These mechanisms include immunosuppressive microenvironment induction, stimulation of tumor growth and regeneration, as well as progression of epithelial-to-mesenchymal transition (Fig. 4).

## Immune suppression

Our recent research demonstrated the important role of the FGF19–FGFR4 signaling pathway in remodeling the immunosuppressive microenvironment of HCC.^124^^,^^125^ The signaling cascade of FGF19–FGFR4 up-regulates the transcription factor, E26 transformation-specific (ETS) variant 4 (ETV4), by engaging the ERK1/2–ELK1 pathway. Overexpressed ETV4 up-regulated the expression of programmed death-ligand 1 (PD-L1) and C–C motif chemokine ligand 2 (CCL2) in HCC cells, leading to an increase in the infiltration of CCR2^+^ tumor-associated macrophages and myeloid-derived suppressor cells, while hindering the infiltration of CD8^+^ T cells. Moreover, we observed a positive feedback loop in the FGF19–FGFR4–ETV4 axis. ETV4 stimulated by FGF19–FGFR4 can also increase FGFR4 expression transcriptionally, resulting in sustained activation of this signaling pathway, ultimately leading to HCC metastasis.^124^ Likewise, our previous study attests to the engagement of FGF19–FGFR4 in the disruption of HCC’s immune microenvironment. FGF19–FGFR4 activation up-regulates CXCL1 and FGFR4 expression by transactivating HOXB5 via the PI3K/Akt/hypoxia-inducible factor 1-alpha (HIF-1α) pathway, leading to the recruitment of C-X-C motif chemokine receptor 2-positive (CXCR2^+^) myeloid-derived suppressor cells in HCC.^125^ The combined approach of the FGFR4 inhibitor BLU-554 (fisogatinib) and the CXCR2 inhibitor SB265610 almost completely eradicated HCC metastasis induced by homeobox B5 (HOXB5).^125^ Furthermore, we noted that the FGF19–FGFR4 signaling pathway up-regulates the expression of SRY-box transcription factor 18 (SOX18) through the p-FRS2/p-GSK3β/β-catenin pathway, thus promoting the metastasis of HCC.^126^ The up-regulation of SOX18 increases the expression of fms-related tyrosine kinase 4 (FLT4), promoting cancer cell invasion and metastasis.^127^ Additionally, SOX18 up-regulation enhances the expression of FGFR4.^126^ BLU9931, another selective FGFR4 inhibitor, significantly suppresses the growth of HCC metastasis induced by SOX18 ^126^.

## Cell survival and self-renewal

In addition, researchers have found that ER stress-induced FGF19 up-regulation can activate the FGFR4–GSK3β–nuclear factor erythroid 2 related factor 2 (Nrf2) signaling cascade to provide cell protection against ER stress.^128^ The transcription factor Nrf2 enhances the expression of the anti-apoptotic gene Bcl-2 and the antioxidant gene HO-1, leading to a considerable decrease in ER stress-induced apoptosis associated with ROS.^128^ Silencing FGF19 offsets the resistance of tumor cells against apoptosis.^128^ The FGF19 pathway is also linked to the self-renewal of liver cancer stem cells, a critical factor for tumor initiation, recurrence, and resistance to therapy. Sequential activation of the FGFR4/SOCE/NFATc2 pathway by FGF19 transcribes and activates genes related to stem cells, maintaining the self-renewal of liver cancer stem cells and thereby contributing to tumor initiation and growth.^129^

## Epithelial-to-mesenchymal transition

FGF19 and FGFR4 play a significant role in the epithelial-to-mesenchymal transition of HCC. The activation of the janus kinase 2 (JAK2)/STAT3 signaling pathway by FGF19–FGFR4 in HCC cells results in the overexpression of epithelial-to-mesenchymal transition-related transcription factors such as Zeb1, Snail, Slug, and Twist. Moreover, STAT3 activation causes down-regulation of the epithelial marker E-cadherin and up-regulation of the mesenchymal marker N-cadherin.^130^ The pivotal function of the IL-6/STAT3 pathway in promoting FGF19-mediated hepatocarcinogenesis in murine models holds potential implications for understanding the pathophysiology of HCC.^131^ STAT3 phosphorylated by FGF19–FGFR4 signaling needs to enter the nucleus with the help of Src.^132^ In addition to this, FGF19-FGFR4 can also contribute to the progression of epithelial-to-mesenchymal transition by activating the Wnt/β-catenin pathway. The activation of this pathway leads to β-catenin stabilization and its accumulation in the nucleus by phosphorylating GSK3β. Nuclear β-catenin suppresses the expression of E-cadherin to promote epithelial-to-mesenchymal transition by increasing the expression of Snail1 and Twist.^133^ This process is also evident in HCC associated with lipid metabolic disorder.^134^

## Targeted therapeutic strategies

The available evidence suggests that interrupting the FGF19–FGFR4 signaling may serve as an effective approach to curbing the proliferation of HCC. Next, the review will initially present an overview of therapeutic approaches that directly target the FGF19–FGFR4 signaling pathway. Additionally, the latest findings concerning synergistic therapy with additional pharmaceutical agents in conjunction with the FGF19–FGFR4 signaling cascade will be reviewed. Finally, the summary will encapsulate the role and impact of the FGF19–FGFR4 signaling pathway on the first-line treatment drugs currently used in HCC.

## Targeting FGF19

Both *in vitro* and *in vivo* studies demonstrated that the monoclonal anti-FGF19 antibody disrupts the binding of FGF19 to FGFR4, thereby suppressing FGF19-mediated tumorigenic activity. Moreover, it prevented colorectal cancer tumor growth in xenografts and significantly curbed the progression of HCC in both FGF19 transgenic and nude mouse models.^116^^,^^135^ The FGF19-derived peptide I0 was developed using computational simulations and labeled with the NIRF dye MPA. Able to bind specifically to FGFR4-positive HCC cells for precise intraoperative tumor identification and resection.^136^

## Targeting FGFR4

Higher levels of FGF19 in tumors serve as reliable biomarkers for selective inhibitors of FGFR4.^119^^,^^120^ Since 2009, the research and development of FGFR4 inhibitors have been continuously progressing. Several FGFR inhibitors are being evaluated in various stages of clinical trials to assess their efficacy and safety in treating HCC (Table 1). These inhibitors target specific isoforms of FGFR, intending to inhibit tumor cell growth and proliferation while minimizing potential toxicity to normal cells. While initial findings are encouraging, the durability and safety profiles of these compounds necessitate additional clinical trials for validation.

**Table 1:** Therapeutic inhibition of FGFR4 in hepatocellular carcinoma.

Name of drug	Conditions	Drugs combined	Phase	NCT number
*FGFR pan-inhibitor*				
AZD4547	Advanced solid malignancies	None	Phase 1	NCT01213160
AZD4547	Advanced solid malignancies	None	Phase 1	NCT00979134
AZD4547	Refractory malignant solid neoplasm	None	Phase 2	NCT04439240
JNJ-42756493 (Erdafitinib)	Advanced malignant solid neoplasm	None	Phase 2	NCT03210714
JNJ-42756493 (Erdafitinib)	Advanced solid tumor	None	Phase 2	NCT04083976
JNJ-42756493 (Erdafitinib)	Carcinoma, hepatocellular	None	Phase 1/2	NCT02421185
BGJ398 (Infigratinib)	Advanced malignant solid neoplasm	None	Phase 2	NCT04233567
BGJ398 (Infigratinib)	Advanced solid tumor	None	Phase 1/2	NCT05222165
BGJ398 (Infigratinib)	Advanced solid tumors with alterations of FGFR1–3	None	Phase 1	NCT01004224
BGJ398 (Infigratinib)	Solid tumor	None	Phase 2	NCT02160041
BGJ398 (Infigratinib)	Solid tumor	None	Phase 2	NCT05019794
BGJ398 (Infigratinib)	Tumor with alterations of the FGFR	None	Phase 1	NCT01697605
PRN1371	Solid tumors	None	Phase 1	NCT02608125
*Combination*				
JNJ-42756493 (Erdafitinib)	Neoplasm	JNJ-63723283	Phase 1	NCT03547037
BGJ398 (Infigratinib)	Advanced solid tumor	BYL719	Phase 1	NCT01928459
BGJ398 (Infigratinib)	Liver cancer	Atezolizumab/Bevacizumab	Phase 1	NCT05510427
LY2874455	Advanced cancer	Phosphate Binders	Phase 1	NCT01212107
*FGFR4 selective inhibitor*				
ABSK-011 (Irpagratinib)	Advanced liver cancer	None	Phase 1	NCT04906434
BLU-554 (Fisogatinib)	Hepatocellular carcinoma	None	Phase 1	NCT02508467
BLU-554 (Fisogatinib)	Hepatocellular carcinoma	None	Phase 1/2	NCT04194801
H3B-6527	Hepatocellular carcinoma	None	Phase 1	NCT02834780
SY-4798	Advanced solid tumor	None	Phase 1	NCT05498519
*Combination*				
ABSK-011 (Irpagratinib)	Hepatocellular carcinoma	Atilizumab	Phase 2	NCT05441475
ABSK-011 (Irpagratinib)	Hepatocellular carcinoma	ABSK043	Phase 2	NCT06978933
FGF401	Hepatocellular carcinoma	PDR001	Phase 1/2	NCT02325739

The vast majority of FGFR pan-inhibitors, including LY2874455, AZD4547, and BGJ398 (infigratinib), are more active against FGFR1–3 and less active against FGFR4.^4^ However, this does not mean that the development of FGFR pan-inhibitors makes no sense. In a study by Tao et al, it was found that there is functional redundancy between FGFR3 and FGFR4 in carcinogenesis, and that FGF19-FGFR3-KLB binding is equally oncogenic. Inhibition of FGFR3 re-sensitized FGF19-positive HCC to the selective FGFR4 inhibitor.^137^

In the development of selective inhibitors of FGFR4, FGFR4 possesses two cysteine residues, Cys477 and Cys552, while Cys522 is exclusive to FGFR4 and absent in other FGFR members. Therefore, selective FGFR4 inhibitors can be attained by targeting the unique cysteine 552 on the FGFR4 hinge region.^138^ Following this principle, FGFR4 selective inhibitors, including BLU9931, BLU-554, and FGF401, have been developed. H3B-6527 was first reported in 2017 as an irreversible selective covalent FGFR4 inhibitor.^139^ Analysis of 40 HCC cell lines and mouse patient-derived xenograft models indicated that elevated levels of FGF19 could serve as an individual prognostic indicator for the sensitivity to H3B-6527 ^139^. Clinical trials of the oral FGFR4 inhibitor ABSK-011 (irpagratinib) for the treatment of HCC are currently recruiting participants (NCT04906434/NCT06978933).

BLU9931 and BLU-554 (fisogatinib) are highly selective and potent irreversible inhibitors of FGFR4. BLU9931 was identified in 2015 as the first FGFR4-selective molecule used to manage HCC with abnormal FGFR4 signaling. Its effectiveness has been demonstrated in tumors with intact FGF19, FGFR4, and KLB signaling pathways.^140^ Our previous study in mouse models demonstrated that BLU9931 had a beneficial impact on inhibiting the growth and metastasis of HCC *in vivo*.^126^ BLU-554, the optimized version of BLU9931, demonstrated noteworthy anti-tumor effects such as inhibition of lung metastasis, relief of immune disorders, and extension of overall survival in the orthotopic transplantation HCC mouse model.^124^^,^^125^ BLU-554 has already been evaluated in phase I clinical trials in patients with advanced HCC (NCT02508467). In this trial, the drug exhibits good tolerability in HCC patients. Moreover, BLU-554 efficacy was 17% in FGF19-positive versus 0% in FGF19-negative patients, correlating FGF19 expression with treatment response.^141^ FGF19 overexpression was defined as a predictor of BLU-554 therapeutic response.^141^^,^^142^ However, researchers have found that patients undergoing treatment with BLU-554 develop mutations in the gatekeeper (V550) or hinge-1 (C552) residues of FGFR4.^142^ These mutations result in some patients who initially respond to BLU-554, but eventually develop progressive disease because of mutation-mediated acquired resistance to BLU-554 ^142^. To overcome BLU-554 resistance, next-generation FGFR4 inhibitor design centers on two strategies.^143^ One counters gatekeeper mutations by designing novel scaffolds to avoid steric clash, as exemplified by FGF401 (roblitinib).^144^ The other focuses on Cys552, developing reversible covalent,^145^^,^^146^ or optimized irreversible covalent inhibitors to improve the efficacy–safety profile.^147^

FGF401 is an effective, selective, and first-rate reversible covalent kinase inhibitor for FGFR4.^148^ The selective inhibition of FGFR4 is facilitated by the formation of a transient covalent bond between the inhibitor and the receptor, resulting in reversible covalent binding. This process also relies on the specific interaction between the 2-formylamino-4-hydroxymethylpteridine moiety of FGF401 and the cysteine at position 552 within the kinase region of FGFR4.^144^ In the preclinical experiments, FGF401 demonstrates significant anti-tumor activity in both orthotopic and ectopic models, as well as xenograft models derived from patients harboring positive expressions of FGF19, FGFR4, and KLB.^119^^,^^144^ The administration of FGF401 resulted in a potent inhibition of tumor growth and proliferation in mouse models. It also initiated programmed cell death and enhanced vascular normalization. Moreover, this intervention markedly extended the overall survival of mice harboring tumors with elevated FGF19 expression.^119^ The molecule’s exceptional selectivity and anti-tumor properties have led to it being the first FGFR4 inhibitor to undergo clinical trials. Currently, it has already completed the phase I or II study titled NCT02325739. In this clinical trial, FGF401 exhibited a favorable pharmacokinetic profile, with good oral absorption and a manageable safety profile.^149^ This trial bequeaths an open question, as no definitive biomarker was pinpointed to reliably forecast the efficacy of FGF401.

## Combined treatment strategy

As summarized above, the FGF19–FGFR4–KLB signaling pathway has been proven to be a promising, precise target for the treatment of HCC, making this pathway an essential focus of HCC treatment in the future. The current treatment approach for HCC mainly relies on the use of multi-kinase inhibitors and immunotherapy. The combination of FGFR4 inhibitors with these two drug classes appears to be a rational approach to improve response rates and delay the development of drug resistance in HCC treatment. Next, we provide a summary of current preclinical and clinical trials that investigate the therapeutic benefits of the combined use of FGFR4 inhibitors and immunotherapy.

Combination BGJ398/FGF401 resulted in a reduction in tumor progression and metastatic spread to the lungs, along with an increase in survival rates in mice with orthotopic tumors. Furthermore, this combination strategy induced immune cell infiltration and programmed cell death, enhancing the therapeutic antitumor impact.^150^

Utilization of immune checkpoint modulators, especially those targeting the PD-1/PD-L1 and cytotoxic-T-lymphocyte-antigen-4 (CTLA-4) pathways, marks a notable progression in HCC therapy. Recent investigations have suggested that adding anti-PD-L1 augmented the antitumor activity of BLU-554. This enhancement is realized by curbing the excessive expression of PD-L1 and the chemokine CCL2 in HCC cells, which in turn restricts the buildup of tumor-associated macrophages and myeloid-derived suppressor cells, and boosts the CD8^+^ T cell infiltration, culminating in the attenuation of HCC spread.^124^

To date, only one combination therapy has undergone clinical trials. In this trial, the combination therapy of spartalizumab (PDR001, an anti-PD-1 antibody) and FGF401 exhibited safety, tolerability, and efficacy similar to single-agent FGF401. However, the combination treatment group had a limited number of patients, which made it difficult to obtain more accurate overall survival information and efficacy conclusions (NCT02325739).^149^

In addition to the combination therapy, studies have explored the potential of combining FGF401 with chemotherapy drugs and other targeted therapies. For example, FGF401 exhibits synergistic effects when used in combination with the microtubule-depolymerizing agent vinorelbine. Such effects include not only further inhibition of tumor growth and promotion of apoptosis but also elongation of survival times in mice carrying FGF19-high tumors, without any signs of increased toxicity.^119^

## FGF19–FGFR4 and first-line HCC treatment drugs

### Sorafenib

HCC patients have been benefiting from sorafenib, a multi-kinase inhibitor, for over a decade. Despite its success, drug resistance often hinders its usage. Sorafenib induces apoptosis in HCC cells by producing ROS, a process that is impeded by increased FGF19 expression or over-activated FGF19–FGFR4 signaling.^129^^,^^151^ Conversely, reducing FGF19 or FGFR4 expression, or treating with ponatinib, enhances ROS production and apoptosis in sorafenib-resistant HCC cells. Targeting FGF19–FGFR4 can partially reverse sorafenib resistance in HCC cells.^151^ Another brief study concluded that the overexpression of the FGF19 pathway led to the reduction of nitric oxide production induced by sorafenib and increased the growth of HCC cells. In contrast, silencing of FGF19 or knockout of FGFR4 rendered HCC cells sensitive to sorafenib treatment by increasing nitric oxide production.^152^ As shown in these studies, an increase in serum levels of FGF19 correlates with poor response to sorafenib treatment and reduced progression-free and overall survival rates.^129^^,^^151^^,^^153^ FGFR4 can also mediate sorafenib resistance independent of FGF19 stimulation. FGFR4, along with EGFR, has been recognized as a target of miR-486-3p, a microRNA that is decreased in sorafenib-resistant cells. Cells with decreased levels of miR-486-3p have increased proliferation and reduced sensitivity to sorafenib.^154^

### Lenvatinib

Lenvatinib targets several kinases, including KIT, RET, platelet-derived growth factor receptor-α, FGFR, and vascular endothelial growth factor receptor. It has demonstrated efficacy in treating unresectable HCC. Patients treated with lenvatinib in the phase III REFLECT trial had similar overall survival to those treated with sorafenib, but improved progression-free survival, time to progression, and objective response rate.^155^

In HCC xenograft models overexpressing FGF19, lenvatinib had an inhibitory effect on the FGF signaling pathway and was accompanied by an inhibition of phosphorylation of FRS2 (a substrate of FGFR1–4).^156^ In contrast, sorafenib had no inhibitory effect on this pathway.^156^ Lenvatinib increased the ferroptosis in Hep3B and HuH7 cells by inhibiting FGFR4 and down-regulating the production of glutathione peroxidase 4 and system X_c_^–^ (xCT).^157^ During immune checkpoint blockade therapy, inhibition of PD-1 on T cells surrounding tumor cells leads to increased expression of PD-L1 on tumor cells and increased Tregs exerting immunosuppressive effects around the tumor, thus affecting efficacy.^158^ Lenvatinib inhibited the FGFR4/STAT5 axis in CD4^+^ Foxp3^+^ T cells, thus hindering their conversion to Tregs, and down-regulated PD-L1 on tumor cells via FGFR4–GSK3β, thus enhancing the efficacy of immune checkpoint blockade against HCC. These effects were reversed by exogenous FGF19 administration.^158^ HCC patients can be screened by FGFR4 expression level and Tregs infiltration for lenvatinib plus anti-PD-1 therapy to achieve better therapeutic effects.^157^^,^^158^ In summary, lenvatinib has an inhibitory effect on the FGF signaling pathway in HCC tumor cells and peripheral immune cells, thus playing a role in the treatment of HCC. Notably, lenvatinib prevented the growth of HCC by blocking tumor microangiogenesis, a mechanism that occurs independently of FGF19–FGFR4 signaling.^156^^,^^159^

In contrast to sorafenib resistance, the level of tumor FGF19 is associated with lenvatinib susceptibility, and lenvatinib selectively eliminates FGF19-expressing tumors. Inhibiting FGF19 effectively eliminates the susceptibility to lenvatinib. Also, down-regulation of FGF19 is observed in lenvatinib-resistant HCC cell lines, and overexpression of FGF19 restores lenvatinib resistance.^160^ However, the article also noted that there is no clear correlation between serum FGF19 levels and tumor FGF19 levels in HCC patients. Therefore, serum FGF19 levels cannot be used to predict the effectiveness of lenvatinib in treating HCC patients.^160^ The Phase III REFLECT Study reported that the increases in FGF19 levels after lenvatinib administration were associated with a positive objective response rate. However, baseline FGF19 levels did not correlate with longer overall survival in tumor patients.^161^ Nevertheless, a retrospective, single-center study involving 27 patients with HCC found that the baseline levels of FGF19 and angiopoietin-2 can serve as reliable indicators of the success of lenvatinib treatment.^162^ This necessitates the use of larger clinical samples and more stringent inclusion criteria to validate the findings.

### The expanding therapeutic landscape of FGFs in liver diseases

Members of the FGF family demonstrate tremendous potential in the treatment of liver diseases, though with distinct emphases.

FGF1 demonstrates multifaceted potential in the treatment of liver diseases. Long-term therapy with recombinant FGF1 can inhibit hepatic glucose production while reducing liver steatosis without causing weight gain.^163^ Engineered FGF1 variants can separate glucose-lowering activity from mitogenic activity.^163^ The non-mitogenic variant FGF1^△HBS^ exerts multiple hepatoprotective effects by activating the FGFR4 signaling pathway on hepatocytes. It not only promotes the nuclear translocation of Nrf2 to enhance antioxidant capacity but also activates the AMP-activated protein kinase (AMPK) pathway, collectively ameliorating steatohepatitis and fibrosis.^164^ Furthermore, long-term administration of FGF1^△HBS^ reduces hepatic BA accumulation.^165^

FGF4 plays a complex dual role in liver diseases, and its function is highly dependent on the disease context. FGF4 acts as an endogenous protective factor in ALD, cholestasis, MASLD, and immune-mediated liver injury via FGFR4 activation. In ALD, it promotes estrogen-related receptor gamma (ERRγ) degradation to suppress cytochrome P450 family 2 subfamily E member 1 (CYP2E1)-mediated toxicity.^166^ In cholestasis, FXR-induced FGF4 inactivates liver receptor homolog-1 (LRH-1), inhibiting BA synthesis.^167^ In MASLD, it enhances lipid oxidation via the Ca^2+^/calmodulin-dependent protein kinase kinase beta (CaMKKβ)–AMPK pathway, reducing steatosis.^168^ In immune-mediated liver injury, FGF4 activates the CaMKKβ/PTEN-induced kinase 1 (PINK1)/Bcl-X_L_ pathway to suppress hepatocyte apoptosis and immune imbalance, mitigating liver injury.^169^ Conversely, genomic amplification of FGF4 drives HCC progression, correlating with aggressive phenotypes.^118^^,^^170^

FGF21 demonstrates multi-faceted hepatoprotective effects through distinct mechanisms. It centrally activates sympathetic output to inhibit hepatic *de novo* lipogenesis and directly targets hepatocytes to reduce cholesterol.^171^ In MASH, FGF21 targets serine/threonine phosphatase PPP6C (the catalytic subunit of protein phosphatase 6) to inhibit mechanistic target of rapamycin complex 1 (mTORC1), promoting transcription factor binding to IGHM enhancer 3 (TFE3)/Lipin1 nuclear translocation.^172^ It also ameliorates cholestatic injury by activating the FGFR4–JNK pathway to inhibit BA synthesis.^173^ Beyond metabolic regulation, FGF21 protects against hepatic ischemia-reperfusion injury by suppressing arachidonic acid metabolism and inflammation,^174^ while preoperative carbohydrate loading induces hepatic FGF21 via protein dilution to confer surgical stress resistance.^175^ Notably, FGF21 reduces alcohol consumption in primates by targeting KLB-expressing neurons in the basolateral amygdala,^176^ and accelerates recovery from ethanol intoxication through norepinephrine activation.^177^ FGF21 analogs (*e.g.*, efruxifermin, pegozafermin) demonstrate significant efficacy in improving liver fibrosis and steatosis in MASH patients with F2/F3 fibrosis, supported by multiple Phase II trials.^178^, ^179^, ^180^ These agents show acceptable safety profiles, with predominantly mild-to-moderate gastrointestinal adverse events and no drug-induced liver injury or deaths.^179^^,^^181^^,^^182^ However, efficacy in patients with compensated cirrhosis remains uncertain.^183^ A GLP-1/FGF21 dual agonist further highlights potential synergistic benefits for metabolic liver diseases.^184^

Both FGF19 and FGF1 produce similar systemic metabolic improvements through the same neuroendocrine mechanism, namely inhibition of the HPA axis.^185^ Both FGF19 and FGF4 carry the risk of causing cancer. Compared with FGF19, FGF4 acts locally through autocrine/paracrine mechanisms, avoiding systemic effects,^185^ and enabling faster feedback inhibition in cholestasis.^167^ The newly discovered role of endogenous FGF4 as an early response factor to counterbalance immune-mediated apoptosis further underscores its function as a local tissue-protective cytokine.^169^ However, the therapeutic development of engineered variants remains more advanced for FGF19 than for FGF4. Compared with FGF19, FGF21 offers a broader therapeutic window without mitogenic/carcinogenic risks, making it advantageous for long-term MASH management. Meanwhile, FGF21 holds greater potential in the treatment of ALD. However, compared with other FGF family members, FGF19 retains its unique and irreplaceable role in regulating BA metabolism.

### Perspectives

**Figure 5:**
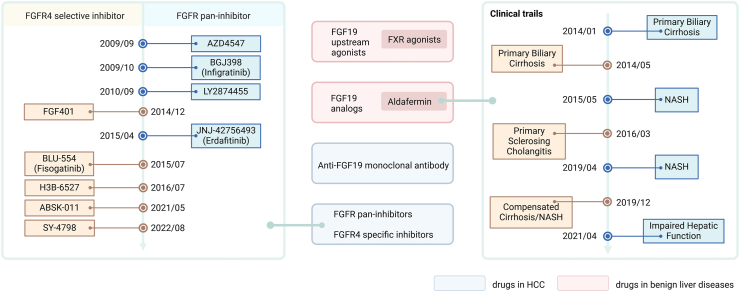
Drugs targeting FGF19–FGFR4 in liver diseases. There are four major drugs targeting the FGF19–FGFR4 signaling pathway in the treatment of liver diseases, including FGF19 upstream agonists, FGF19 analogs, anti-FGF19 monoclonal antibodies, and FGFR inhibitors. The most important upstream agonist of FGF19 is the FXR agonist. The most rapidly studied FGF19 analog is aldafermin. FGFR inhibitors are the most studied in hepatocellular carcinoma. The left timeline shows a milestone in the development of FGFR inhibitors. The right timeline shows the milestones of aldafermin in clinical trials of various liver diseases.

At present, the research in the field of liver therapy targeting the FGF19–FGFR4 signaling pathway presents two development directions: one is to inhibit FGFR4 in HCC, and the other is to increase the expression of FGF19 in a variety of benign liver diseases. This trend can be observed in the development of related drug research. In the treatment of HCC, there are few studies on FGF19 monoclonal antibodies.^135^ The main research direction is to develop FGFR4 inhibitors, and the development of highly specific and highly selective receptor inhibitors has become an obvious trend (Fig. 5). In terms of activating this pathway, a variety of FGF19 upstream FXR agonists^186^^,^^187^ and FGF19 analog aldafermin have been applied in the treatment of various benign liver diseases (Fig. 5).

The therapeutic potential of wild-type FGF19 is constrained by its associated oncogenic risk. The development of non-tumorigenic FGF19 variants has followed two main directions. One strategy involves completely altering receptor preference. The variant FGF19-7, by replacing partial sequences, shifts its target from hepatic FGFR4 to adipose tissue FGFR1c, thereby improving glucose homeostasis while completely avoiding hepatocyte proliferation risk.^11^ Combined mutagenesis of the N-terminal amino acid residues (38–42) and the heparin-binding domain of FGF19 can achieve similar effects.^33^ However, this development strategy also forfeits the benefits associated with activating the FGFR4 metabolic pathway. The other strategy focuses on the selective activation of FGFR4 signaling. Variants such as aldafermin and FGF19–M52, through N-terminal deletions and point mutations, effectively inhibit metabolic pathways like CYP7A1 while failing to activate proliferative signals such as STAT3.^188^^,^^189^ Mechanistic studies indicate that reducing FGFR4 dimerization potential by weakening FGF19’s binding ability to its receptor, coreceptor, or heparan sulfate preferentially eliminates mitogenic activity while preserving metabolic function.^190^^,^^191^ Protein engineering has laid the foundation for developing next-generation therapies for metabolic liver diseases.

In mouse models, elevated FGF19 has been demonstrated to function as a beneficial compensatory mechanism that inhibits BA synthesis in ALD and cholestatic diseases.^90^^,^^95^ However, in human patients, FGF19 is frequently observed to be ectopically expressed under such pathological conditions, with its sources including hepatocytes, cholangiocytes, and mast cells.^98^^,^^99^^,^^192^ This expression may be driven by specific inflammatory cytokines. These factors are often abundant in the local microenvironment, such as IL-6 and tumor necrosis factor alpha (TNF-α).^193^ Alternatively, it could be a direct cellular response to endogenous or exogenous stress. The accumulation of toxic BAs may also trigger this response. Elucidating the upstream triggers of this expression is crucial. It is key to distinguishing the pathophysiological mechanisms of these diseases from MASLD.

Serum FGF19 levels are significantly elevated in patients with advanced disease or poor prognosis, suggesting its potential as a biomarker for assessing liver disease severity and predicting outcomes.^194^ In cases of severe cholestasis, increased serum FGF19 accompanied by decreased CYP7A1 and C4 levels does not indicate preserved metabolic regulatory function; rather, it reflects severe impairment of hepatocyte function and synthetic failure.^194^ Furthermore, FGF19 can activate hepatic stellate cells via the FGFR4–JAK2–STAT3 signaling pathway, promoting their polarization into inflammatory cancer-associated fibroblasts and enhancing neutrophil infiltration and NET formation through up-regulation of factors such as IL-1α, thereby exacerbating disease progression.^195^ The FGF19–FGFR4–angiopoietin-like 4 (ANGPTL4) axis has been identified as a key mechanism driving hepatic stellate cell activation and the formation of a liver metastatic niche.^196^

These findings indicate a clear disease stage-dependent nature of the metabolic benefits mediated by the FGF19–FGFR4 signaling pathway. Future studies should focus on two key objectives. First, the specific mechanisms of ectopically expressed FGF19 need to be elucidated. Second, the precise stages at which this signaling exerts protective effects within the liver immune microenvironment must be defined. Addressing these questions will provide a critical foundation for developing precision therapies for related liver diseases.

## Conclusions

This article systematically reviews the regulation of the FGF19–FGFR4 signaling pathway on the function of the normal liver and the significance of its abnormal changes in benign liver diseases. In HCC, excessive FGF19–FGFR4 activity modifies the tumor microenvironment, promoting cancer cell survival, metastasis, and chemoresistance. FGF19–FGFR4 exhibits either protective or detrimental effects depending on the type and progression of the liver diseases. This complicates therapeutic strategies, requiring a balance between efficacy and safety. Therapies targeting this pathway, including FGFR4 inhibitors and FGF19 analogs, are currently in clinical trials. However, challenges remain, such as the risk of metabolic disruption and the induction of HCC with long-term use. Further research is essential to refine FGF19–FGFR4 targeted therapies, develop more specific inhibitors and agonists, and explore synergistic combination treatments. Identifying FGF19–FGFR4-associated biomarkers for treatment response and elucidating the interplay with other pathways will advance personalized approaches to liver disease management.

## CRediT authorship contribution statement

**Zhangfan Wu:** Writing – review & editing, Writing – original draft, Visualization, Project administration. **Yijun Wang:** Writing – review & editing, Writing – original draft, Visualization. **Jiaqian Zhang:** Writing – review & editing, Writing – original draft. **Siwen Li:** Writing – review & editing, Writing – original draft, Visualization. **Junjie Wen:** Writing – review & editing. **Dian Hu:** Writing – review & editing. **Junqing Jiang:** Writing – review & editing. **Zerui Zhang:** Supervision, Conceptualization. **Xiangyuan Luo:** Supervision, Conceptualization. **Limin Xia:** Supervision, Funding acquisition, Conceptualization.

## Funding

This work was supported by grants from the 10.13039/501100001809National Natural Science Foundation of China (No. U23A20451, 82273310 to L.X.), the 10.13039/501100003819Natural Science Foundation of Hubei Province, China (No. 2022CFA016 to L.X.), and the Basic Research Support Program of Huazhong University of Science and Technology (China) (No. 2023BR038 to L.X.).

## Conflict of interests

The authors declared no competing interests.
